# 3-phase dual-energy CT scan as a feasible salvage imaging modality for the identification of non-localizing parathyroid adenomas: a prospective study

**DOI:** 10.1186/s40463-015-0098-y

**Published:** 2015-10-31

**Authors:** Michael Roskies, Xiaoyang Liu, Michael P. Hier, Richard J. Payne, Alex Mlynarek, Veronique Forest, Mark Levental, Reza Forghani

**Affiliations:** Department of Otolaryngology-Head and Neck Surgery, Jewish General Hospital & McGill University, Montreal, Quebec Canada; Department of Radiology, Jewish General Hospital & McGill University, Montreal, Quebec Canada; Segal Cancer Centre and Lady Davis Institute for Medical Research, Jewish General Hospital & McGillUniversity, Montreal, Quebec Canada

**Keywords:** Parathyroid adenoma, Head and neck surgery, Computed tomography, Dual-energy CT, 4D-CT, 4DCT, Minimally invasive parathyroidectomy

## Abstract

**Objectives:**

Accurate pre-operative imaging of parathyroid adenomas (PAs) is essential for successful minimally invasive surgery; however, rates of non-localizing PAs can be as high as 18 %. Multiphasic dual-energy CT (DECT) has the potential to increase accuracy of PA detection by enabling creation of paired material maps and spectral tissue characterization. This study prospectively evaluated the utility of 3-phase DECT for PA identification in patients with failed localizatio n via standard imaging.

**Methods:**

Patients with primary hyperparathyroidism and non-localizing PAs underwent a 3 phase post-contrast DECT scan acquired at 25, 55, and 85 s. The scans were prospectively evaluated by two head and neck radiologists. Pre-operative localization was compared to intraoperative localization and final histopathology. A post-hoc DECT spectral density characterization was performed on pathologically-proven PAs.

**Results:**

Out of 29 patients with primary hyperparathyroidism and non-localized PAs, DECT identified candidates in 26. Of the 23 patients who underwent parathyroidectomy, DECT provided precise anatomic localization in 20 patients (PPV = 87.0 %), one with multi-gland disease. The virtual unenhanced images were not found to be useful for diagnosis but successful diagnosis was made without an unenhanced phase regardless. Spectral analysis demonstrated a distinct spectral Hounsfield attenuation curve for PAs compared to lymph nodes on arterial phase images.

**Conclusion:**

3-phase DECT without an unenhanced phase is a feasible salvage imaging modality for previously non-localizing parathyroid adenomas. Optimal interpretation is achieved based on a combination of perfusion characteristics and other morphologic features. Advanced spectral DECT analysis has the potential for further increasing accuracy of PA identification in the future.

## Background

Accurate pre-operative localization of parathyroid adenomas (PAs) is essential for successful minimally invasive surgery. At many institutions, including ours, this is done based on two concordant studies. Typically, the most common approach for PA localization is by a combination of sestamibi and ultrasound [1, 2]. However, despite their popularity, these techniques have certain pitfalls. Limitations of sestamibi studies for parathyroid adenoma identification include absent radiotracer retention in some adenomas, diminishing sensitivity with decreasing adenomatous tissue, and potential confounding by concurrent thyroid disease or the occasional hot thyroid nodule [2]. Sestamibi also has low sensitivity for multiglandular disease. Ultrasound is operator dependent and, in addition, locations where ectopic PAs typically are located such as deep within the neck, the retropharyngeal space, and the mediastinum, tend to be areas that are blind-spots for ultrasound [2]. Reported sensitivities for the ability to lateralize (localize) PAs to the correct side of the neck are approximately 57 to 88 % for ultrasound and 65 to 86 % for sestamibi [3]. The addition of SPECT or SPECT/CT may further increase sensitivity for PAs to approximately 90 % or more according to some studies [2, 4], but that has not been the experience in our institution.

4-dimensional CT (4D-CT) is increasingly used for localization of PAs [3, 5–10]. 4D-CT enables characterization of perfusion characteristics of candidate PAs. The main principle behind 4D-CT is that PAs have different perfusion characteristics compared to lymph nodes and normal thyroid gland [3, 5]. In its original form, 4D-CT included a non-contrast acquisition followed by three post-contrast acquisitions that include an arterial phase (usually at 25 s) with two additional scans obtained after variable delays [2, 3, 5]. In general, PAs have more rapid and greater arterial phase enhancement and a more rapid rate of contrast wash-out compared to the normal thyroid gland [5]. Lymph nodes are typically hypoenhancing compared to PAs on arterial phase images, but demonstrate slow progressive enhancement on more delayed images, also a pattern different from typical PAs [5]. The combination of perfusion characteristics and high spatial resolution of CT technique accounts for the success of 4D-CT [5], with some studies reporting accuracy for lateralization of 94 % [5]. As a result, there is increasing interest and use of 4D-CT for PA identification and localization. However, one of the concerns about 4D-CT is radiation exposure because of multiple acquisitions. To this end, there are reports demonstrating that not all of the phases described in the original 4D-CT protocol may be necessary for accurate PA localization [11, 12]. Whereas one approach is to simply eliminate one or more phases from conventional multiphasic CT, another approach is to use more advanced techniques such as dual-energy CT for increasing the diagnostic yield and therefore potentially reducing the number of acquisition needed for a diagnostic exam.

Dual energy CT (DECT) is an advanced CT technique that evaluates tissues at different X-ray energies, enabling spectral evaluation and material tissue characterization beyond what is possible with conventional CT [13–16]. Normally, the attenuation of different tissues and materials varies when scanned at high and low tube voltages, depending on their specific elemental properties. With DECT, projection data are typically obtained simultaneously or near-simultaneously at 80 and 140 kVp (kilovolt peak) [14]. Using sophisticated computer algorithms, the data at different acquisition energies can then be normalized to specific combinations of two reference materials, such as iodine, water or calcium. Furthermore, the spectral data can be used to generate image sets at different predicted energy levels (keV; kiloelectron volts), referred to as virtual monochromatic images (VMI). As such, DECT enables generation of virtual unenhanced images as well as other advanced tissue characterization not possible with conventional CT, all done by post-processing and without the need for any additional scan acquisitions. There are emerging applications of DECT in all of the major subspecialties in radiology [16–22]. In the head and neck, there is increasing evidence that DECT can improve visualization of head and neck squamous cell carcinoma and increase accuracy for evaluation of thyroid cartilage invasion, among other applications [13, 22–28].

Currently, there are only isolated reports of DECT for localization of PAs [29] but no systematic evaluation of this technique. In this study, we prospectively evaluated the utility of multiphasic DECT for PA localization in a group of patients having discordant or unidentified PAs on a workup consisting at a minimum of ultrasound and sestamibi. A 3-phase DECT, without an unenhanced phase, was performed with the ability to create virtual unenhanced images as needed if needed for diagnostic evaluation. This was followed by a post-hoc spectral density evaluation of PAs and lymph nodes.

## Methods

### Patients

The study was approved by the institutional review board at the Jewish General Hospital. In the period from September 2013 to April 2014, after obtaining consent, we recruited all patients with primary hyperparathyroidism and non-concordant imaging studies (Table 1). At our institution, the standard studies used for PA localization are ultrasound and sestamibi SPECT/CT and all patients had undergone these studies. However, some patients had undergone additional investigations, including MRIs (15/29) and seven patients who had undergone a total of nine negative surgical explorations (Table 1). Non-concordance was defined as either unidentified (i.e. standard imaging fails to identify any PA) or discordant (i.e. standard imaging does not agree on the location). Demographic data was recorded and patients were divided into “unidentified” or “discordant” groups. Patients with a history of iodine allergy were excluded from the study.

**Table 1:** Patient population and clinical presentation (*p* > 0.05 for all demographic data)

	Discordant	Unidentified
Subjects included	18	11
Average age	63.61	54.36
F:M ratio	12:6	7:4
Symptoms		
Incidental hypercalcemia	14	8
Osteopenia/porosis	2	2
Renal failure	2	1
Previous workup		
Ultrasound	22	11
Sestamibi	23	20
MRI	10	5
Surgical exploration	5	4

### CT technique

All patients were scanned with the same 64-section dual-energy scanner (GE Discovery CT750HD; GE Healthcare, Milwaukee, WI). Scans were obtained at 25, 55, and 85 s after injection of 100 mL of iopamidol at 3.5 mL/s. The 25 and 55 s acquisitions were acquired in dual-energy rapid 80–140-kVp switching mode using the gemstone spectral imaging protocol [13]. These were acquired with a GSI preset 1, with a large scan field of view (up to 50 cm), 40-mm beam collimation, 0.6-second rotation time, and 0.984:1 helical pitch, resulting in a maximal tube current of approximately 640 mA. Images were reconstructed into 1.25 mm sections with 25-cm display field of view and 512 × 512 matrix. 70 keV VMIs, the VMI believed to simulate the standard 120 kVp single energy acquisition by extrapolation from abdominal CT studies, were reconstructed and transferred to PACS for interpretation. Source spectral images were transferred to a dedicated workstation (GE Advantage workstation 4.6; GE Healthcare, Milwaukee, WI) where virtual unenhanced image reconstruction or more advanced spectral analysis could be performed.

### Prospective PA identification

The scans were prospectively reviewed by one of two attending head and neck radiologists with 5 (R.F.) and 15 (M.L.) years post-fellowship experience in head and neck radiology. Primary interpretation and prospective localization of PAs was performed using the multiphasic 70 keV VMIs. If needed, additional virtual unenhanced images were generated to help image interpretation at the discretion of the reporting radiologist. If virtual unenhanced images were used to assist interpretation, this was recorded. If patient was called back for additional imaging, such as to obtain true unenhanced images, this was also recorded. Potential candidate adenomas were described based on their size, shape, presence of an identifiable supplying artery (referred to as polar artery), and exact anatomic location with respect to the thyroid gland and associated cartilages. Depending on ability to localize a potential adenoma, the study was termed “DECT positive” or “DECT negative”.

### Surgical confirmation

The imaging findings were compared with localization during minimally invasive surgery and histopathologic confirmation. Sensitivity was calculated for pre-operative identification of correct side and quadrant. Successful surgical excision was considered based on histopathology and a decrease in the level of blood serum parathyroid hormone of greater than 50 % post-operatively.

### Post-hoc advanced DECT characterization

Since little is known about the spectral characteristics of PAs, a post-hoc analysis of spectral curves of a subset of PAs [13] was performed and compared to lymph nodes in order to evaluate for potential differences in their spectral characteristics. Analysis was performed on the dedicated GE Advantage workstation (4.6; GE Healthcare, Milwaukee, WI). Quantitative image analysis was performed using region of interest (ROI) analysis. Scans were retrospectively reconstructed into different VMI energy levels ranging from 40 to 140 keV in 5 keV increments. PA and lymph node evaluation was performed by measuring mean CT attenuation (in Hounsfield units; HU) ± standard deviation (SD) within regions of interest (ROIs) across the entire range of VMI energy levels. All ROIs were placed by an attending head and neck radiologist (R.F.). ROIs were placed on the homogenous enhancing part of PAs or lymph nodes, excluding any heterogenous or cystic foci within the PA if present. Care was also taken not to overlap with adjacent tissues in order to avoid volume averaging with other tissues. Because of frequently small size of PAs and lymph nodes, small ROIs had to be used. However, to obtain a representative sample, 3 ROIs were obtained in each structure and the mean attenuation of the 3 ROIs calculated at each energy level for each structure. Each ROI was large enough to cover the enhancing area without overlap with heterogenous or cystic internal foci or adjacent tissue. For lymph nodes, normal lymph nodes were selected, avoiding areas obscured by artifact. When possible, nearby nodes (level VI or IV) were selected. If those were too small for analysis, then a level IB or IIA node was selected for analysis. The average area for each individual ROI used was 5.62 mm^2^ (range 1.05–10.15 mm^2^).

### Statistical analysis

Positive predictive value was calculated for PA identification and final pathology in the unidentified and discordant studies. For quantitative ROI analysis, results were reported as mean ± SD. Spectral Hounsfield attenuation curves were generated from 40 to 140 keV, in 5 keV increments for comparison of PAs and LNs. For each structure (PA or lymph node), the average density was determined by calculating the average of the three ROIs used per structure in that patient. Data from different patients were then pooled at each keV for comparison of PAs with lymph nodes. Comparison of means was performed using an unpaired two-tailed *t*-test. A *p*-value less than 0.05 was considered to be statistically significant. We used Graphpad Prism version 6.005 for statistical analysis (GraphPad Software, La Jolla California USA, www.graphpad.com, GraphPad Software, Inc., La Jolla, CA).

## Results

### Patient population and clinical presentation

In total, 29 patients were evaluated in this study, 11 in the unidentified and 18 in the discordant groups (Table 1). The average age of participants was 60.1 years old (range 39–76), consisting of 19 women and 10 men. The most common presenting complaint was asymptomatic incidental hypercalcemia with elevated parathyroid hormone, but presentations ranged from osteopenia to renal failure (Table 1). Total counts for imaging/procedures performed prior to DECT included: 33 ultrasounds, 43 sestamibi scans, 15 MRIs, and nine previous exploratory procedures (on seven patients).

### Prospective parathyroid adenoma identification and surgical outcome

Multiphasic dual-energy CTs localized potential PAs in 26 of 29 patients: 10/11 in the equivocal and 16/18 in the discordant group. One patient in the latter group had two candidate adenomas identified, corresponding to a 94.4 % “DECT positive” rate overall. Of the 26 DECT positive studies, 23 patients have undergone minimally invasive parathyroidectomy at this time and 20 operations were successful (PPV 87.0 %). Both adenomas in the patient with bilateral disease were histologically positive corresponding to 21 adenomas total and an 87.5 % PPV overall. Of the seven patients with previous negative surgical explorations, DECT found candidate adenomas in six. Operations were successful (positive localization and pathology) in four of the six patients. Of the three studies in which the DECT identified candidate could not be confirmed surgically, two were from the discordant and one from the unidentified group.

**Fig. 1:**
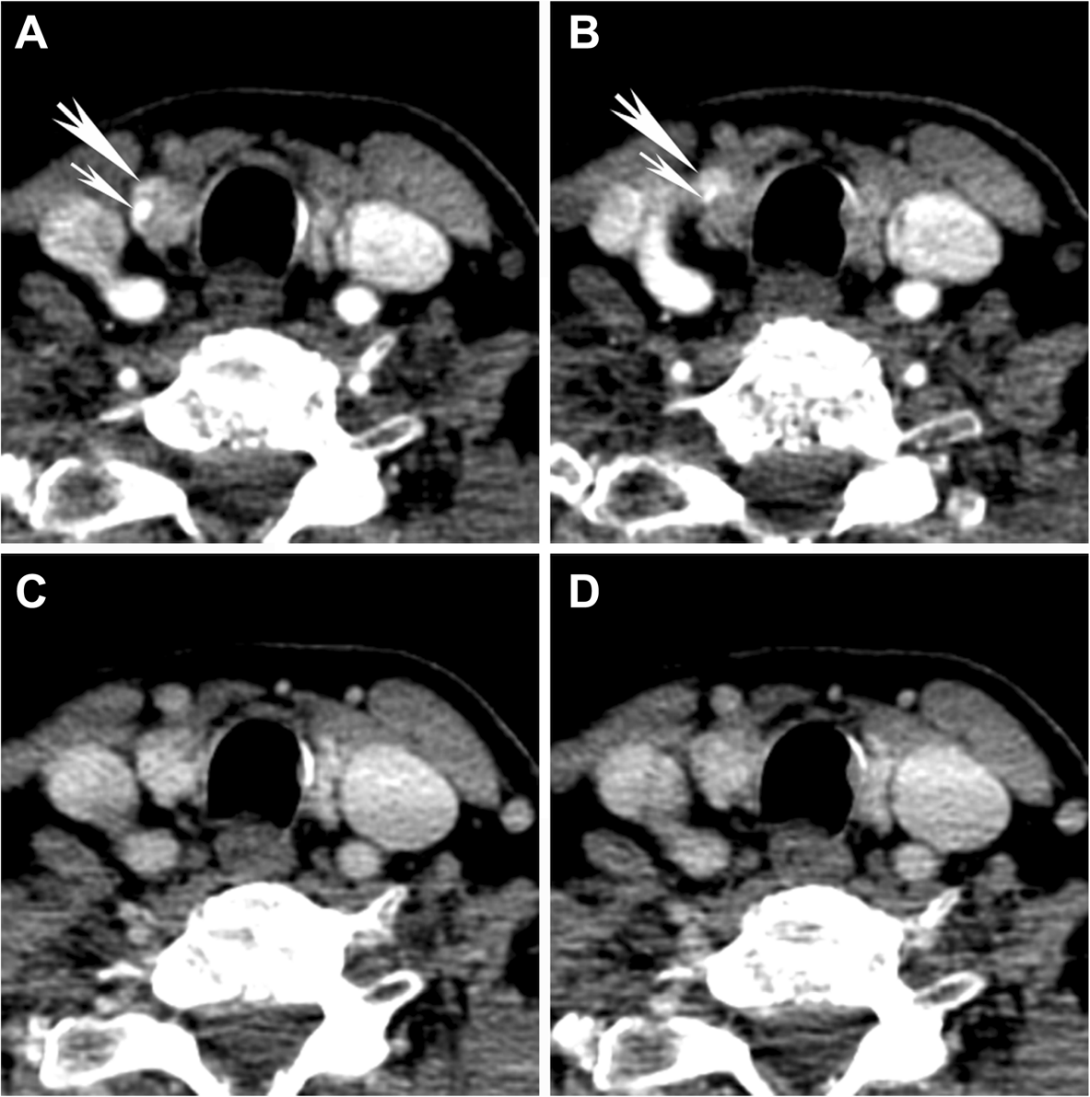
Typical perfusion characteristics in an intrathyroidal parathyroid adenoma. 70 keV VMIs at (**a**, **b**) 25 s and (**c**, **d**) 55 s are shown of a surgically and pathologically proven intrathyroidal parathyroid adenoma (*large arrow*). The feeding vessel supplying the adenoma is also seen (*small arrow*) and is helpful for diagnosis, sometimes referred to as the polar artery. There is the typical rapid and robust enhancement on arterial phase images (**a**, **b**). On the more delayed images, there is contrast washout from the adenoma but increased attenuation of the thyroid gland and the adenoma cannot be clearly distinguished from the thyroid gland (**c**, **d**)

**Fig. 2:**
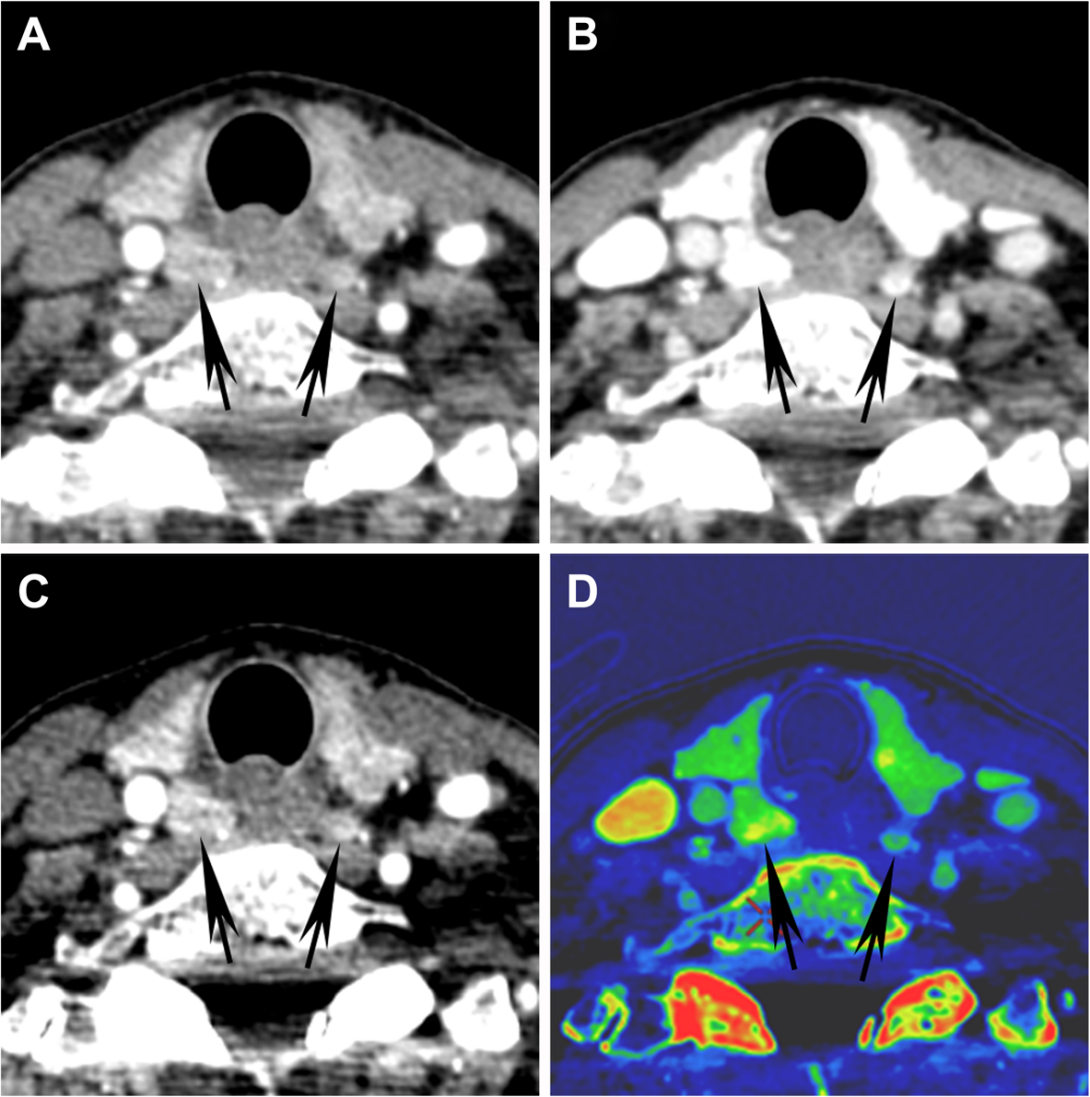
Atypical perfusion characteristics in bilateral parathyroid adenomas. (**a**) 25 s 70 keV VMI, (**b**) 55 s 70 keV VMI, (**c**) 25 s 50 keV VMI, and (**d**) 25 s iodine overlay map are shown demonstrating surgically and pathologically proven bilateral parathyroid adenomas (*arrows*). In this case, a typical robust arterial phase enhancement with rapid washout is not shown (adenoma attenuation on the 25 s images was less than 100 HU). However, the presence of a fat plane separating the adenomas from the thyroid gland, location, and different appearance from normal lymph nodes enabled a confident pre-operative diagnosis in this case. The 50 keV VMI (**c**) is shown as an example of how DECT low energy reconstructions can accentuate the density of enhancing/iodine containing structures (compare **c** to **a**). DECT also enables creation of iodine overlay maps (**d**), highlighting the iodine content of tissues and enabling a quantitative estimation of tissue iodine content

**Fig. 3:**
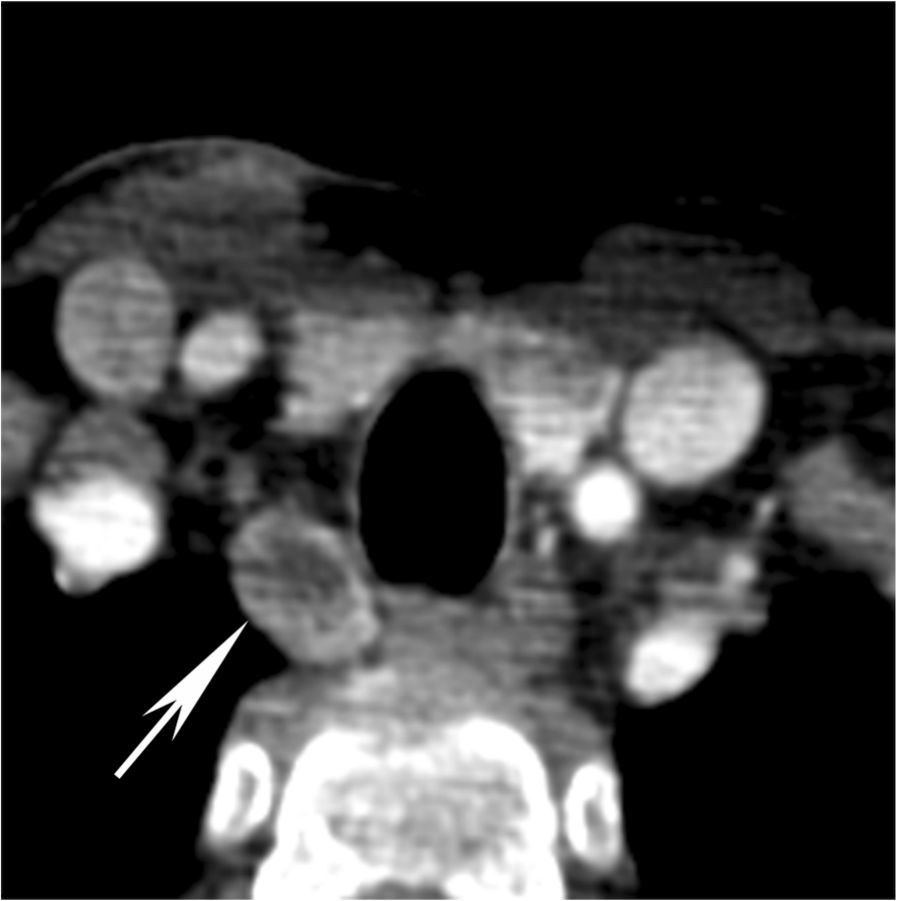
Parathyroid adenoma with cystic internal change. Example of surgically and pathologically proven parathyroid adenoma (*arrow*) extending to the right tracheo-esophageal groove

Among the 20 patients with pathologically proven PAs, DECT was concordant with the sestamibi SPECT/CT in seven cases but US in only one case. Basic characteristics of PAs are summarized in Table 2 and the location of the PAs in the discordant and unidentified groups is summarized in Table 3. Sizes ranged from 0.6 to 2.7 cm and means were similar in the two groups (1.43 cm discordant vs. 1.24 cm unidentified). Perfusion characteristics were a key component of PA identification, particularly on the 25 s arterial phase images (Fig. 1). However, not all PAs demonstrated typical robust arterial phase enhancement or rapid washout and as such, other features were also important in identifying and localizing a PA (Table 2, Figs. 2 and 3). These included features that allowed confident separation of the PA from the thyroid gland, such as presence of fat plane between the PA and thyroid, perfusion pattern distinct from thyroid gland, and other morphologic characteristics enabling reliable distinction from lymph nodes (Table 2).

**Table 2:** Basic characteristics of PAs on DECT

Shape (%)	
Oval	64.3
Tear drop	21.4
Triangular	7.1
Tubular	7.1
Margins (%)	
Smooth	78.6
Lobulated	21.4
Other defining characteristics	
Low attenuation component (%)	28.6
Fat plane separating from thyroid (%)	85.7
Polar artery present (%)	92.6
Location (%)	
Perrier [37]	
Type A (4.8)	
Type B (9.5)	
Type C (23.8)	
Type D (28.6)	
Type E (4.8)	
Type F (19.0)	
Type G (9.5)

**Table 3:** Location of PAs in the discordant and equivocal groups

	Pathologically-proven (*N* = 21)	
DECT characteristics	Discordant	Unidentified
Positive	14	7
Location (% of total (21); Perrier classification [37])	A (4.8)	A (0)
	B (9.4)	B (0)
	C (18.9)	C (4.8)
	D (14.3)	D (14.3)
	E (0)	E (4.8)
	F (14.3)	F (4.8)
	G (4.8)	G (4.8)

### Advanced DECT analysis

**Fig. 4:**
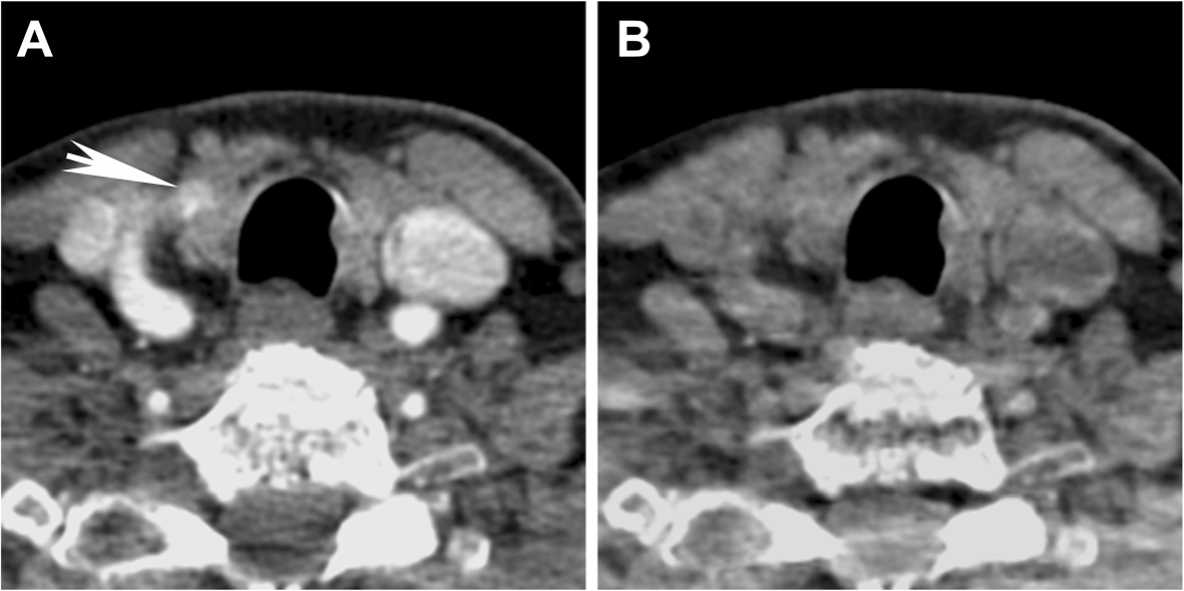
Virtual unenhanced DECT images. **a** 70 keV VMI and (**b**) virtual unenhanced image of the intrathyroidal parathyroid adenoma in Fig. 1 are shown. The parathyroid adenoma seen on the 70 keV VMI (*arrow*) cannot be seen on the virtual unenhanced image (**b**) because of suppression of iodinated contrast on that image. The iodinated contrast in the vessels as well as iodine within the thyroid gland are also suppressed

In the 29 patients evaluated here, the radiologists created virtual unenhanced images in only two of the cases and these were deemed not to be helpful. The limitation of virtual unenhanced images for PA localization is that in addition to the enhancing PA, the intrinsic iodine content of the thyroid gland is also suppressed (Fig. 4). As such, DECT virtual unenhanced images cannot be used as a complete substitute for the unenhanced CT for purposes of PA characterization. Early during recruitment, two out of 29 patients were called back in order to obtain actual unenhanced images. On retrospective evaluation, it was felt that these did not add significantly to the study and no patients were recalled for obtaining unenhanced CT during the work-up of the latter 20 patients in this study.

**Fig. 5:**
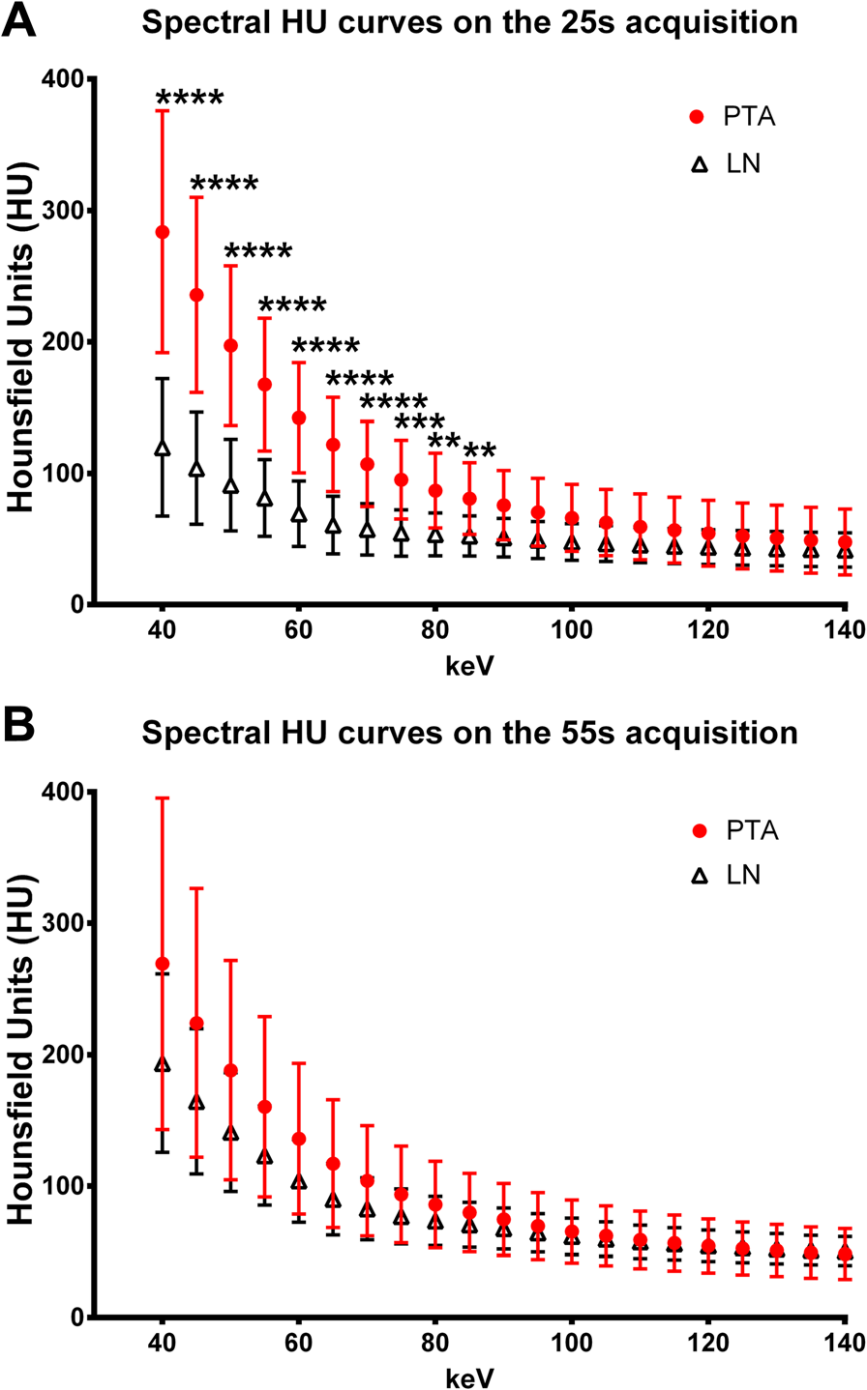
Spectral Hounsfield unit (HU) curve analysis of parathyroid adenomas compared to lymph nodes. Spectral HU analysis of 13 normal appearing lymph nodes (LN) and 14 surgical and pathologically proven adenomas (PA) from 13 patients are shown from the (**a**) 25 s and (**b**) 55 s DECT acquisitions. PA have different spectral HU characteristics compared to LN on the 25 s but not the 55 s acquisition with density separation on the left (lower energy) side of the curve. **P < 0.01, ***P < 0.001, ****P < 0.0001

As part of this pilot study using DECT, a post-hoc quantitative spectral Hounsfield unit attenuation curve analysis was performed, comparing characteristics of PAs to lymph nodes (Fig. 5). On the 25 s arterial acquisition, there was a significant difference between the spectral attenuation curves of PAs compared to lymph nodes, with density separation in the low energy range (*P* < 0.01 - *P* < 0.0001; Fig. 5). Although there was a trend for density separation in the lower energy range on the 55 s acquisition, this was not statistically significant (Fig. 5).

## Discussion

Primary hyperparathyroidism (PHPT) is most commonly caused by a solitary benign parathyroid adenoma and the treatment is surgical excision [30]. In order to limit the extent of dissection in searching for the pathologic gland, preoperative localization studies are used [31]. These studies currently include sestamibi scanning, ultrasonography, computed tomography (CT), magnetic resonance imaging (MRI), positron emission tomography (PET) and angiography [2, 3, 32–34]. Pre-operative concordant images have a dramatic impact on success and associated morbidity of minimally invasive surgery [35].

There is increasing popularity of 4D-CT for localization of PAs although there are concerns about the radiation exposure associated with the classic 4 phase 4D-CT [3, 5–10]. While the effective dose of the typical 4D-CT protocol is greater than that of scintigraphy, studies have shown the lifetime incidence of cancer compared to baseline cancer risk for this population is negligible for either study [36]. Despite this and in order to minimize radiation exposure from the procedure to the extent possible, some groups are decreasing the number of acquisitions [11, 12]. DECT scans can be used to create virtual unenhanced images or iodine overlay maps that can estimate the iodine content of a structure based on a single acquisition [14, 15]. In addition, virtual monochromatic images can be created at different energy levels, and these can be evaluated quantitatively, potentially increasing the analytic capabilities of CT technique [13–15]. This could potentially improve accuracy and in turn enable a reduced number of acquisitions.

In this investigation, we used a 3 phase CT technique, with DECT acquisitions, to localize unidentified or discordant PAs prospectively. Consistent with other studies [3, 5–12], multiphasic CT was effective in localizing PAs in a significant percentage of cases, including cases of multi-gland disease and intrathyroidal PA. Although the radiologists had the ability to generate and use virtual unenhanced images, this was overall deemed not necessary and after the use for two cases in the early part of the study, without benefit, these were not created or used for the other cases. The problem of using virtual unenhanced images for PA identification is that both the iodinated contrast in the enhancing PA and the intrinsic iodine within the thyroid gland are suppressed (Fig. 4), defeating the purpose of the reconstructions for distinction of PA from thyroid tissue. On the other hand, we also demonstrate successful identification of PAs prospectively without the need for an unenhanced scan. During the early part of the study, two patients were called back for an unenhanced study. However, in retrospect these were deemed not necessary and were not performed in any of the patients later on. This is consistent with more recent reports demonstrating successful “4D-CT” with a reduced number of phases [11, 12].

Although our investigation did not reveal a role for the DECT virtual unenhanced images for PA localization, post-hoc spectral Hounsfield unit attenuation curve analysis demonstrated a difference in the characteristics of PAs compared to lymph nodes on arterial phase images (Fig. 5). Arterial phase images are one of the most important acquisitions in 4D-CT performed for PA localization and these preliminary observations suggest that DECT can further increase accuracy during this phase of the exam, which may in turn enable further reduction of the number of acquisitions without decreasing diagnostic accuracy in the future. Other potential applications of DECT could be improved PA visualization on low energy virtual monochromatic images and the use of estimated iodine content for PA identification (Fig. 2). These are topics of great interest for future research.

Although the perfusion characteristics are central in identifying PAs, other features such as location, presence of feeding or polar artery, and other characteristics that help distinguish PAs from normal lymph nodes such as cystic internal change were also important for accurate identification of PAs (Table 2). Furthermore, not all of the PAs demonstrated a typical robust arterial phase enhancement with rapid wash-out (Fig. 2). Therefore, rather than focusing on absolute thresholds, it may be more important to identify combinations of features that help distinguish PAs from potentially mimicking normal structures. Of course, it is possible that we observed a higher frequency of atypical appearing PAs because the study was used to evaluate unidentified or discordant PAs, resulting in a selection bias.

In this study, we were able to localize previously unidentified parathyroid adenomas in 26 of 29 patients. Twenty three of these patients have undergone surgical exploration at this time, and DECT correctly identified 21 PAs in 20 of those patients. We also demonstrate a high success-rate of minimally invasive parathyroidectomy on primary cases and even some secondary cases. Among the three false positives, two were in patients with prior surgery. Therefore, one must at least consider the possibility that these PAs may have not been found because of extensive scarring from the patient’s prior surgery. One of the strengths of this study is that all PA identification was done prospectively. The limitation is that the numbers are relatively small. As many of these were outside referrals, another limitation could be that not all of the standard imaging was done at the institution were DECT was performed, potentially introducing a bias. However, among the 20 patients who successfully underwent surgery, 13 had sestamibi and 12 US at the same institution and therefore the proposed bias could not account for the success of DECT in these cases. In addition, a small number of patients could not be analyzed because they have not undergone surgery yet (either because of loss to follow-up or surgical wait-list time). Nonetheless, our results demonstrate the feasibility of a multiphasic study without an unenhanced phase and promising results for DECT spectral analysis for improving the diagnostic evaluation of PAs. The impact of more advanced DECT analysis will have to be tested in larger and ideally prospective use of these characteristics in future studies.

## Conclusion

In this prospective study, we demonstrate that a 3 phase CT technique, with DECT acquisitions and without an unenhanced phase, has high accuracy in identifying previously unidentified or discordant PAs. Furthermore, our post-hoc analysis demonstrates significant differences in the spectral characteristics of PAs compared to lymph nodes on arterial phase images. This suggests that advanced DECT analysis has the potential to further increase accuracy for PA identification, which could potentially enable a reduction in the number of CT acquisitions and associated radiation exposure. This is an interesting topic for future research.

## Abbreviations

PA: Parathyroid adenoma
CT: Computed tomography
4D-CT: 4-dimensional CT
DECT: Dual-energy CT
PPV: Positive predictive value
VMI: Virtual monochromatic images
ROI: Region of interest
kVp: kilovolt peak
keV: kiloelectron volts

## References

[1] Munk RS, Payne RJ, Luria BJ, Hier MP, Black MJ (2008). Preoperative localization in primary hyperparathyroidism. J Otolaryngol Head Neck Surg.

[2] Phillips CD, Shatzkes DR (2012). Imaging of the parathyroid glands. Semin Ultrasound CT MR.

[3] Rodgers SE, Hunter GJ, Hamberg LM, Schellingerhout D, Doherty DB, Ayers GD (2006). Improved preoperative planning for directed parathyroidectomy with 4-dimensional computed tomography. Surgery.

[4] Patel CN, Salahudeen HM, Lansdown M, Scarsbrook AF (2010). Clinical utility of ultrasound and 99mTc sestamibi SPECT/CT for preoperative localization of parathyroid adenoma in patients with primary hyperparathyroidism. Clin Radiol.

[5] Hunter GJ, Schellingerhout D, Vu TH, Perrier ND, Hamberg LM (2012). Accuracy of four-dimensional CT for the localization of abnormal parathyroid glands in patients with primary hyperparathyroidism. Radiology.

[6] Hunter GJ, Ginat DT, Kelly HR, Halpern EF, Hamberg LM (2014). Discriminating parathyroid adenoma from local mimics by using inherent tissue attenuation and vascular information obtained with four-dimensional CT: formulation of a multinomial logistic regression model. Radiology.

[7] Kelly HR, Hamberg LM, Hunter GJ (2014). 4D-CT for preoperative localization of abnormal parathyroid glands in patients with hyperparathyroidism: accuracy and ability to stratify patients by unilateral versus bilateral disease in surgery-naive and re-exploration patients. AJNR Am J Neuroradiol.

[8] Hoang JK, Sung WK, Bahl M, Phillips CD (2014). How to perform parathyroid 4D CT: tips and traps for technique and interpretation. Radiology.

[9] Mortenson MM, Evans DB, Lee JE, Hunter GJ, Shellingerhout D, Vu T (2008). Parathyroid exploration in the reoperative neck: improved preoperative localization with 4D-computed tomography. J Am Coll Surg.

[10] Chazen JL, Gupta A, Dunning A, Phillips CD (2012). Diagnostic accuracy of 4D-CT for parathyroid adenomas and hyperplasia. AJNR Am J Neuroradiol.

[11] Welling RD, Olson JA, Kranz PG, Eastwood JD, Hoang JK (2011). Bilateral retropharyngeal parathyroid hyperplasia detected with 4D multidetector row CT. AJNR Am J Neuroradiol.

[12] Noureldine SI, Aygun N, Walden MJ, Hassoon A, Gujar SK, Tufano RP (2014). Multiphase computed tomography for localization of parathyroid disease in patients with primary hyperparathyroidism: How many phases do we really need?. Surgery.

[13] Forghani R, Levental M, Gupta R, Lam S, Dadfar N, Curtin HD (2015). Different Spectral Hounsfield Unit Curve and High-Energy Virtual Monochromatic Image Characteristics of Squamous Cell Carcinoma Compared with Nonossified Thyroid Cartilage. AJNR Am J Neuroradiol.

[14] Johnson T, Fink C, Schönberg SO, Reiser MF (2011). Dual Energy CT in Clinical Practice.

[15] Johnson TR (2012). Dual-energy CT: general principles. AJR Am J Roentgenol.

[16] Pomerantz SR, Kamalian S, Zhang D, Gupta R, Rapalino O, Sahani DV (2013). Virtual monochromatic reconstruction of dual-energy unenhanced head CT at 65–75 keV maximizes image quality compared with conventional polychromatic CT. Radiology.

[17] De Cecco CN, Darnell A, Rengo M, Muscogiuri G, Bellini D, Ayuso C (2012). Dual-energy CT: oncologic applications. AJR Am J Roentgenol.

[18] Heye T, Nelson RC, Ho LM, Marin D, Boll DT (2012). Dual-energy CT applications in the abdomen. AJR Am J Roentgenol.

[19] Lu GM, Zhao Y, Zhang LJ, Schoepf UJ (2012). Dual-energy CT of the lung. AJR Am J Roentgenol.

[20] Postma AA, Hofman PA, Stadler AA, van Oostenbrugge RJ, Tijssen MP, Wildberger JE (2012). Dual-energy CT of the brain and intracranial vessels. AJR Am J Roentgenol.

[21] Vliegenthart R, Pelgrim GJ, Ebersberger U, Rowe GW, Oudkerk M, Schoepf UJ (2012). Dual-energy CT of the heart. AJR Am J Roentgenol.

[22] Forghani R. Advanced dual-energy CT for head and neck cancer imaging. Expert review of anticancer therapy. 2015;In press. 10.1586/14737140.2015.1108193 26535613

[23] Albrecht MH, Scholtz JE, Kraft J, Bauer RW, Kaup M, Dewes P (2015). Assessment of an Advanced Monoenergetic Reconstruction Technique in Dual-Energy Computed Tomography of Head and Neck Cancer. Eur Radiol.

[24] Kuno H, Onaya H, Iwata R, Kobayashi T, Fujii S, Hayashi R (2012). Evaluation of cartilage invasion by laryngeal and hypopharyngeal squamous cell carcinoma with dual-energy CT. Radiology.

[25] Lam S, Gupta R, Levental M, Yu E, Curtin HD, Forghani R (2015). Optimal Virtual Monochromatic Images for Evaluation of Normal Tissues and Head and Neck Cancer Using Dual-Energy CT. AJNR Am J Neuroradiol.

[26] Liu X, Ouyang D, Li H, Zhang R, Lv Y, Yang A (2015). Papillary Thyroid Cancer: Dual-Energy Spectral CT Quantitative Parameters for Preoperative Diagnosis of Metastasis to the Cervical Lymph Nodes. Radiology.

[27] Tawfik AM, Kerl JM, Bauer RW, Nour-Eldin NE, Naguib NN, Vogl TJ (2012). Dual-energy CT of head and neck cancer: average weighting of low- and high-voltage acquisitions to improve lesion delineation and image quality-initial clinical experience. Investig Radiol.

[28] Tawfik AM, Razek AA, Kerl JM, Nour-Eldin NE, Bauer R, Vogl TJ (2014). Comparison of dual-energy CT-derived iodine content and iodine overlay of normal, inflammatory and metastatic squamous cell carcinoma cervical lymph nodes. Eur Radiol.

[29] Gimm O, Juhlin C, Morales O, Persson A (2010). Dual-energy computed tomography localizes ectopic parathyroid adenoma. J Clin Endocrinol Metab.

[30] Eufrazino C, Veras A, Bandeira F (2013). Epidemiology of Primary Hyperparathyroidism and its Non-classical Manifestations in the City of Recife, Brazil. Clin Med Insights Endocrinol Diabetes.

[31] Akbaba G, Berker D, Isik S, Aydin Y, Ciliz D, Peksoy I (2012). A comparative study of pre-operative imaging methods in patients with primary hyperparathyroidism: ultrasonography, 99mTc sestamibi, single photon emission computed tomography, and magnetic resonance imaging. J Endocrinol Invest.

[32] Lumachi F, Ermani M, Basso S, Zucchetta P, Borsato N, Favia G (2001). Localization of parathyroid tumours in the minimally invasive era: which technique should be chosen? Population-based analysis of 253 patients undergoing parathyroidectomy and factors affecting parathyroid gland detection. Endocr Relat Cancer.

[33] Schalin-Jantti C, Ryhanen E, Heiskanen I, Seppanen M, Arola J, Schildt J (2013). Planar scintigraphy with 123I/99mTc-sestamibi, 99mTc-sestamibi SPECT/CT, 11C-methionine PET/CT, or selective venous sampling before reoperation of primary hyperparathyroidism?. J. Nucl. Med..

[34] Herrmann K, Takei T, Kanegae K, Shiga T, Buck AK, Altomonte J (2009). Clinical value and limitations of [11C]-methionine PET for detection and localization of suspected parathyroid adenomas. Mol Imaging Biol.

[35] Bergenfelz AO, Wallin G, Jansson S, Eriksson H, Martensson H, Christiansen P (2011). Results of surgery for sporadic primary hyperparathyroidism in patients with preoperatively negative sestamibi scintigraphy and ultrasound. Langenbecks Arch Surg.

[36] Hoang JK, Reiman RE, Nguyen GB, Januzis N, Chin BB, Lowry C (2015). Lifetime Attributable Risk of Cancer From Radiation Exposure During Parathyroid Imaging: Comparison of 4D CT and Parathyroid Scintigraphy. AJR Am J Roentgenol.

[37] Perrier ND, Edeiken B, Nunez R, Gayed I, Jimenez C, Busaidy N (2009). A novel nomenclature to classify parathyroid adenomas. World J Surg.

